# Evaluation of the degree of liver fibrosis and genetical characteristics in HIV patients with spontaneous clearance of HCV in Cartagena, Spain

**DOI:** 10.7448/IAS.17.4.19637

**Published:** 2014-11-02

**Authors:** Francisco Jesús Vera Méndez, Amaya Jimeno Almazán, Clara Smilg Nicolás, Begoña Alcaraz Vidal, Onofre Martínez Madrid, Mar Alcalde Encinas, Josefina García García, Lorena Martínez Fernández, Pablo Conesa Zamora, Elena Ruiz Belmonte, Paloma Escribano Viñas, Javier Trujillo Santos

**Affiliations:** 1 Internal Medicine, Hospital Santa Lucía, Cartagena, Spain; 2 Molecular Genetics Unit, Hospital Santa Lucía, Cartagena, Spain

## Abstract

**Introduction:**

Single-nucleotide polymorphisms (SNPs) in the region of the Interleukin 28B (IL28B) gene on chromosome 19, coding for the interferon (IFN)-λ3, are involved in hepatitis c virus (HCV), spontaneous clearance. There is little information on the degree of liver fibrosis (LF) in HIV patients, who have had spontaneous clearance of HCV. Our objective in this study is to assess the degree of liver fibrosis in this population, as well as to identify key genetic characteristics associated with spontaneous clearance.

**Materials and Methods:**

Retrospective descriptive study that evaluated from 1 January 2013 to 30 May 2014, the HIV-HCV coinfected patients in which it has been demonstrated spontaneous clearance of HCV infection. The main variables analyzed were (1) genetics: Haplotype determination of genetic polymorphism SNP rs12979860 in the region of IL 28B gene and (2) grade of LF measured in kilopascals (kPa) by transient elastography (TE).

**Results:**

We evaluated 205 patients with HIV and HCV coinfection, of whom, 17 patients (8.3%), presented spontaneous clearance. Fourteen patients (82.4%) had HIV CV<20 copies/mL, while the mean CD4 cell count was 396 (SD: 245) cells/mcL. Nine (53%) patients were analyzed for SNP rs12979860 of IL 28B gene. Nine patients (100%) had the CC haplotypes, and no cases of CT and TT haplotypes were detected (p<0.001). LF was measured by TE in 12 (70.6%) patients. The median of LF (Kpa) was 5.95 (IQR 4.78). In four patients (33.3%) were observed significant LF (F3–F4). In the univariate analysis, no significant differences in the median of LF (Kpa) of HIV patients with spontaneous clearance of HCV were observed with respect to the coinfected HIV-HCV patients with SVR to antiviral therapy (N=34; median 9.6 Kpa; IQR: 10.7; p=0.92) or the coinfected HIV-HCV group who did not receive antiviral therapy (N=124; median 8.5 kPa; IQR: 7.65; p=0.85).

**Conclusions:**

Spontaneous clearance of HCV in our series of patients coinfected with HIV infection represents an uncommon clinical phenomenon. The immune status was preserved and most patients had HIV virological suppression. In all patients in which the IL 28B genetic polymorphism was determined, CC haplotypes were found. The degree of LF (Kpa) in this population was low (<9.5 Kpa) and a low proportion of subjects showed significant LF (F3–F4). No differences in the degree of LF were found in this group compared to coinfected patients receiving HCV treatment with SVR and compared to untreated patients coinfected with HIV-HCV.

